# Importance of mega-environments in evaluation and identification of climate resilient maize hybrids (*Zea mays* L.)

**DOI:** 10.1371/journal.pone.0295518

**Published:** 2023-12-14

**Authors:** Rajashekhar M. Kachapur, Nandan L. Patil, Sidramappa C. Talekar, Mrutyunjaya C. Wali, Gopalakrishna Naidu, Shiddappa R. Salakinakop, Sharanappa I. Harlapur, Jayant S. Bhat, Prakash H. Kuchanur

**Affiliations:** 1 AICRP-Maize, University of Agricultural Sciences, Dharwad, Karnataka, India; 2 Department of Genetics and Plant Breeding, College of Agriculture, Dharwad, Karnataka, India; 3 Former Director of Research, KSNUAHS, Shivamogga, Karnataka, India; 4 AICRP-Soybean, University of Agricultural Sciences, Dharwad, Karnataka, India; 5 ICAR-IARI-Regional Research Center, Dharwad, Karnataka, India; 6 Department of Genetics and Plant Breeding, AC, Bheemrayangudi, UAS, Raichur, Karnataka, India; KGUT: Graduate University of Advanced Technology, ISLAMIC REPUBLIC OF IRAN

## Abstract

Multi-location experiments on maize were conducted from 2016 to 2019 at ten locations distributed across two agro-climatic zones (ACZ) i.e., ACZ-3 and ACZ-8 of Karnataka, India. Individual analysis of variance for each location-year combination showed significant differences among the hybrids; similarly, combined analysis showed a higher proportion of GE interaction variance than due to genotype. Mega-environments were identified using biplot approaches such as AMMI, GGE, and WAASB methodologies for the years 2016 to 2019. The BLUP method revealed a high correlation between grain yield and stability indices ranging from 0.67 to 1.0. Considering all three methods together, the three location pairs Arabhavi-Belavatagi, Bailhongal-Belavatagi, and Hagari-Sirguppa had three occurrences in the same mega-environment with a value of 0.67, and these location combinations consistently produced winning genotypes. Among the common winning genotypes identified, it was G7 during 2016 and 2017 and G10 during 2018 and 2019, based on WAASBY. The likelihood of Arabhavi-Nippani, Hagari-Mudhol, and Dharwad-Hagari occurring in the same mega-environment is minimal because they did not share the same winning genotype, with the exception of a small number of events. Despite being in the same agro-climatic zone, Arabhavi, Hagari, and Mudhol rarely had a winning genotype in common. An agro-climatic zone is grouped based on climatic and soil conditions which doesn’t consider GE interaction of cultivars thus, releasing the cultivars for commercial cultivation considering mega environments pattern would enhance the yield for the given target region.

## Introduction

Maize (*Zea mays* L.) is an important cereal grain crop after rice and wheat around the world and also in India. As compared to rice and wheat, it is widely adapted to varied agro-climatic conditions and is physiologically more efficient in converting the inputs into higher grain yield because of the C_4_ photosynthetic pathway as compared to the C3 pathway in rice and wheat. Maize is a highly cross-pollinated crop, and heterosis has been commercially exploited, resulting in a higher grain yield. In addition, maize responds well to agronomic management practices and is photo-insensitive; hence, it is being cultivated throughout the year (kharif, rabi, and *spring*) in India in a variety of agro-climatic conditions right from mean sea level (MSL) to 3000 above MSL [1]. When maize is cultivated in such ecologically diverse environments, i.e., altitude, rainfall, and soil properties, the production and productivity of maize get affected [2]. It has been reported that more than 50% of the variability in maize yields is due to climatic variability alone [3]. It is practically challenging to expect a maize hybrid to perform consistently across all environments in such a heterogeneous environmental situation. To overcome these problems, climate-resilient maize hybrids that are being bred specifically for improved tolerance to variable and changing climatic conditions and yield potential are needed in the twenty-first century [4].

Maize covers approximately an area of 205.901 million hectare across the globe with average productivity of 5.95 t/ha. India with an area of 9.860 million hectare occupies fourth place and has a productivity of 3.21 t/ha [5]. This significant gap *w*.*r*.*t*. yield indicates vast scope for improvement. The development and use of climate-resilient maize hybrids have already resulted in some success in this direction in Sub-Saharan Africa, where 53 million people in 13 countries have benefited from the development and deployment of climate-resilient maize hybrids [6]. In order to achieve a sustainable maize yield, the first step in this process is to map the most suitable mega-environments (MEs) and then select a suitable maize hybrid for that mega-environment. CIMMYT has initiated a strategy by grouping the world’s maize-producing regions into broad ecologies such as the lowland tropics, subtropics, mid-altitudes, and highlands [7]. Similar attempts were made in India, utilizing a GIS technique for the construction of mega-environments for maize [8,9]. The term "mega-environment" was first used by CIMMYT in 1989 to target the development of wheat germplasm by grouping regions based on shared environmental factors and cropping system requirements [10]. It is described as a portion or region of a crop-producing area for any species of a particular crop, with which it may not be adjacent, having a homogeneous environment that promotes the performance of similar genotypes [8]. Another description of a mega-environment is a group of environments that share the same or best cultivar(s), and in such a mega-environment, the cultivar-location interaction pattern should be repeatable [11], thereby improving heritability and genetic gain, which results in increasing regional productivity [12]. Mega-environments were originally intended to assist crop breeders in reducing genotype and environment interaction, and the information on MEs can also be used in reorganizing the ecological zones for a particular genotype, planning location-specific breeding programmes, avoiding non-performing testing locations, and maximizing resource utilization [13].

The most popular methods for identifying mega-environments are multivariate statistical approaches based on biplot analysis, specifically GGE (genotypic main effect plus genotype by environment interaction) and AMMI (additive main effects and multiplicative interaction) [14,15]. Another complementary method to group locations is by employing major environmental factors such as temperature, rainfall, soil type etc., and then identifying agro-climatic zones based on the environmental factors.

Accordingly, the Indian Institute of Maize Research (IIMR), Ludhiana, has classified the maize-growing areas of India into five major ecologies, such as the Northern Hill Zone (NHZ), the North Western Plain Zone (NWPZ), the North Eastern Plain Zone (NEPZ), the Peninsular Zone (PZ), and the Central Western Zone (CWZ) (www.iimr.icar.gov.in). The maize-growing area of Karnataka state is part of the Peninsular Zone (PZ) of India and is characterized by a tropical environment with 11.0 to 12.5 hours of day length and a mean temperature of > 24° C. Based on the various soil and climatic factors, the agro-climatic conditions of Karnataka further have been classified into 10 zones, each of which has distinct characteristics ranging from very high rainfall (Coastal Zone: Zone-10) to very low rainfall (North Eastern Dry Zone: Zone-2). In Karnataka, maize is grown on more than 1.50 million hectares of land each year in such diverse agro-climatic conditions (www.iimr.icar.gov.in/annualreports). Therefore, it is necessary to divide this vast and varied maize-growing region into various mega-environments. However, classifying all ten zones into different mega environments is a herculean task. Thus, two major agro-climatic zones i.e., northern dry zone (Zone-3) and the northern transition zone (Zone-8) were nearly 40–45% of the maize area of Karnataka state is found and accordingly, the test locations were selected for evaluation with the objective of understanding the genotype-environment interaction (GEI) pattern of the hybrids and identifying stable maize hybrids across target environments, apart from identifying mega-environment(s) for maize cultivation in Northern Karnataka.

## Material and methods

**Fig 1:**
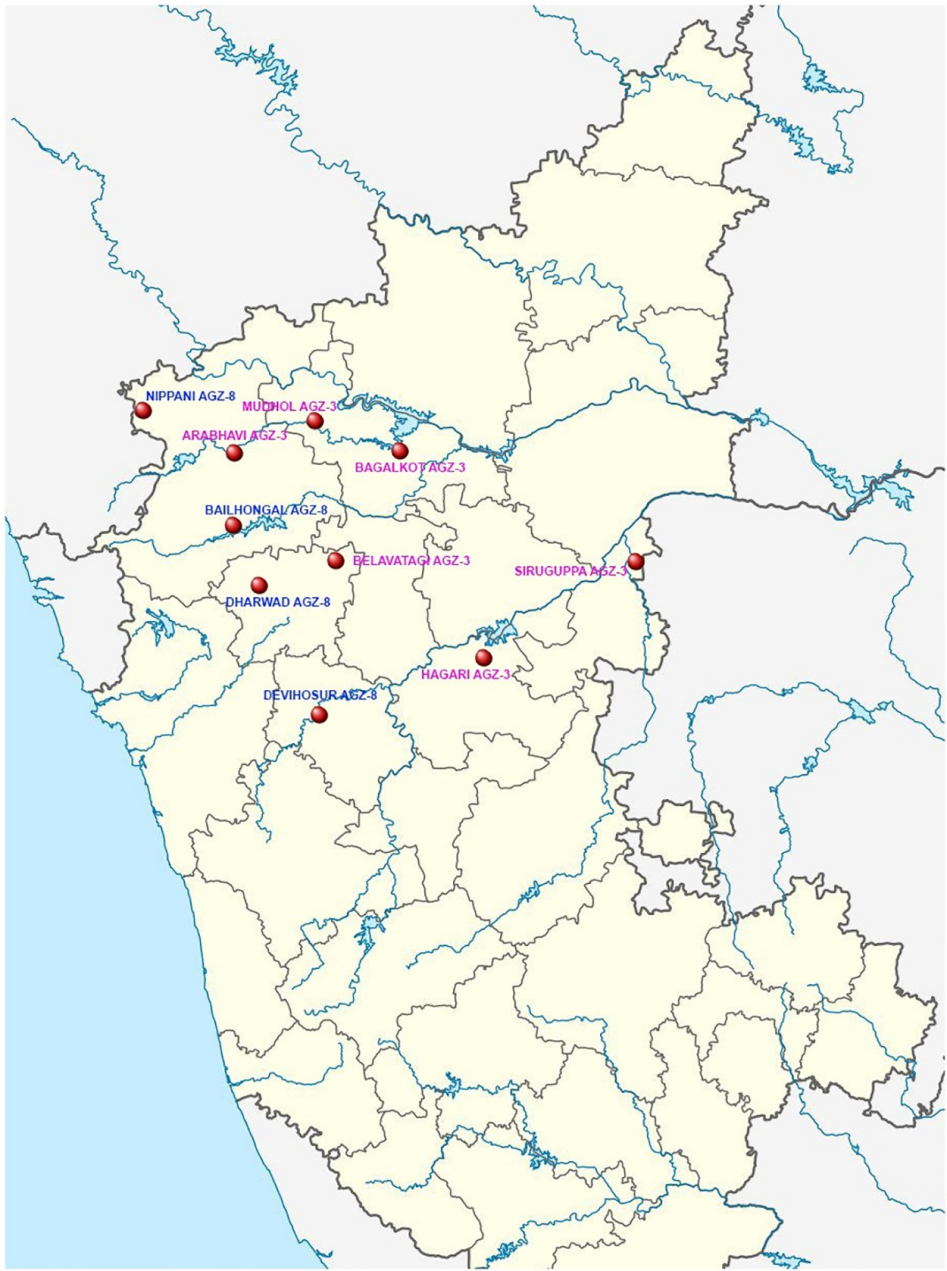
Location of ten test environments spread across Northern Karnataka. Note: Pink and Blue represent agro-climatic zone 3 and 8 respectively. (Source https://commons.wikimedia.org/wiki/File:Political_map_of_Indian_state_of_Karnataka.png).

Multi-environment evaluation trials of maize consisted of promising pre-released maize hybrids of four research institutes namely, University of Agricultural Sciences, Dharwad; University of Agricultural Sciences, Raichur, Indian Agricultural Research Institute, New Delhi and Karnataka State Seed Corporation, Dharwad along with three check hybrids. The experiments were conducted for four years from 2016 to 2019 during Kharif season (June to October) in ten diverse environments located across two agro-climatic zones (AGZ) *i*.*e*., the Northern Dry Zone (Zone 3) and the Northern Transitional Zone (Zone 8) of Northern Karnataka (Tables 1 and 2). A location map and list of hybrids evaluated are presented in Fig 1. The experimental hybrids were evaluated for their mean performance at nine locations in 2016 and eight locations in 2017, 2018, and 2019. The list of hybrids evaluated at various locations for year 2016 to 2019 is presented in S1 Table. All the experiments were laid out in a randomized complete block design (RCBD) with three replications. Each hybrid was grown in four rows of four meters in length by following the spacing of 60 cm between rows and 20 cm between plants. The standard package of practices was followed to raise a healthy crop. To estimate grain yield first the cob weight and moisture content of freshly harvested cobs of each entry was measured. It was further threshed to calculate shelling per cent and dried to reach steady moisture content. Finally, the grain yield per hectare was calculated by adjusting the moisture to 15% [16].

**Table 1:** Agro-climatic location of the experimental sites.

		Zone-3						Zone-8			
Parameters		Arabhavi	Belavatagi	Mudhol	Bagalkot	Hagari	Siruguppa	Dharwad	Nippani	Devihosur	Bailhongal
Longitude		16.11 N	15.57 N	16.33 N	15.85 N	15.15 N	15.61 N	15.25 N	16.40 N	14.79 N	15.81 N
Latitude		74° 52’ E	75° 36’ E	75° 28’ E	75° 77’ E	77°05’ E	76°90’ E	75° 07’ E	74° 38’ E	75°33’ E	74°86’ E
Altitude above MSL (m)		625	609	548	559	415	380	678	585	551	699
GPS co-ordinate		16.22, 74.81	15.59, 75.37	16.33, 75.28	16.16, 75.66	15.15, 77.05	15.62, 76.89	15.49, 74.98	16.40, 74.38	14.78, 75.34	15.82, 74.85
Soil type		Black with sandy loam	Deep black soil	Black soil with sandy loam	Black soil	Black soil	Black soil	Black (medium deep)	Black	Black (medium deep)	Black soil
Annual rainfall (mm)		480	580	511	573	531	550	807	803	700	564
Temperature (°C)	Min (Avg)	20.8	22.4	22.5	22.5	23.2	22.8	19.5	21.4	21.3	20.5
	Max (Avg)	30.8	31.8	32.2	32.1	32.9	33.0	31.5	31.3	31.3	30.1

**Table 2:** Average grain yield of maize hybrids (kg/ha) in Zone 3 and 8 of Karnataka from Kharif 2016 to 2019.

Agro climatic zones	Test locations	Years				Mean Grain yield (kg/ha)
		2016	2017	2018	2019	
Zone 3 (Northern Dry zone)	Arabhavi (ARA)	9126	8621	8804	9115	8916
	Bagalkot (BAG)	-	6362	6536	6580	6492
	Belavatagi (BEL)	4777	-	5912	8854	6515
	Hagari (HAG)	5383	5813	-	5726	5641
	Mudhol (MUD)	4789	6363	7150	8056	6589
	Siruguppa (SIR)	3558	9486	12247	6676	7992
	Mean (Zone-3)	5526	7329	8130	7501	7122
Zone 8 (Northern transition zone)	Bailhongal (BAI)	3878	6763	8956	4209	5951
	Devihosur (DEV)	5436	6862	-	-	6149
	Dharwad (DWR)	11554	-	6951	7629	8711
	Nippani (NIP)	6898	4554	10778	-	7410
	Mean (Zone-8)	6942	6060	8895	5919	6954
	CD at *p* ≤ 0.05	1230	1676	1731	1612	980
	Overall Mean grain yield across zones (kg/ha)	6093	6906	8385	7150	7133

Note: ‘-‘ Trial vitiated for the given year and location.

The replicated data from individual test locations and across locations was compiled, and analysis of variance was carried out for each individual location (individual ANOVA) and combined across locations for a given year (combined ANOVA), with all the factors considered fixed using the *metan* package in the R programme [17].

### Genotype by Environment (GE) interaction analysis

GE interaction is a complex phenomenon in which the response of a cultivar is dependent on external environmental factors. G × E interaction arises due to the differences in the sensitivities of genotypes to the different environmental conditions. A cultivar with high grain yield across locations and minimum GE interaction is preferred by a breeder and is termed as ‘stable cultivar’. To identify stable genotypes and investigate the GE interaction among crops, numerous statistical methods have been proposed since the 19^th^ century, ranging from univariate methods such as the regression method [18,19] and stability method [20] to multivariate methods like factor analytic models, AMMI and GGE. For evaluating multi-location trials, biplot approaches such as GGE, AMMI and WAASB are widely used [21,22] as they provide information on stable genotypes and types of GE interactions and also assist in the identification of mega-environments.

The two-dimensional GGE biplot based on SREG_2_ always uses an intermediate portion of G and GE variation [23,24] between AMMI_1_ and AMMI_2_. Therefore, a GGE biplot should always be close to the best model. Another biplot approach, AMMI [25,26], expresses GE interaction in terms of multiplicative effect, with the first principal component axis retaining the majority of the GEI pattern (IPCA). The AMMI method was earlier employed to identify mega-environments using the Louisiana corn trial [10]. A new technique that combines the AMMI and BLUP methods where GEI effects are reduced through BLUP before being exposed to AMMI analysis [27,28]. Similar to AMMI analysis, the nominal yield is plotted against the environmental IPCA1 scores to identify the mega-environments. In this work, we aimed to identify mega-environments for maize cultivation in the Northern Karnataka region using maize experimental data from the kharif season of 2016 to 2019 across ten locations in agro climatic Zones 3 and 8 (Table 1).

In the first method, environment-centered data was subjected to SVD (singular value decomposition) using GGE biplot analysis and then eigen values were partitioned among the environmental vectors (column metric preserving) to identify relationships among the environments [27,29]. The first two principal components were used to produce the GGE biplot [30–32]. In the second method, the genotype and environment main effects are fitted as additive effects through ANOVA using AMMI analysis [10], and the residual effect, i.e., the GE interaction, is modelled as a multiplicative effect through singular value decomposition (SVD) to obtain IPCs (interaction principal components) [26,32]. In the third method, BLUPs were obtained from effects generated by a linear mixed model, which were further subjected to SVD [27] to estimate genotype and environmental scores.

### Cross-validation procedure

To identify the best model and evaluate the efficiency of AMMI and BLUP models, a cross-validation procedure was performed. The raw dataset was separated into two datasets: training and validation. The training dataset has N-1 replications, i.e., two replications, whereas the validation dataset comprises only one replication. The Root Mean Square Prediction Difference (RMSPD) values were used to select and compare AMMI and BLUP models [33].

### Estimation of stability indices

Stability indices were estimated based on AMMI and BLUP analyses. AMMI-based indices were ASV (AMMI Stability Index), SSI (Simultaneous Selection Index), SIPC, EV (Average of Squared Eigen Values), and ZA (Absolute Value of the Relative Contribution of IPCs to the Interaction). BLUP values of hybrids (S2 Table) were used in the estimation of indices, *viz*., HMGV (harmonic mean of genotypic values), RPGV (relative performance of genotypic values), and HMRPGV (harmonic mean of relative performance of genotypic values).

The new index WAASBY (Weighted Average of Absolute Scores of BLUP and Yield) [26] was used to measure the grain yield performance and stability of genotypes. Here, the best genotype is determined by rescaling and weighing both the grain yield (GY) and WAASB (Weighted Average of Absolute Scores of BLUP), which are rescaled and weighted to identify the best genotype. The weight age for WAASB and GY might range from 100/0 to 0/100. We adopted a 50/50 weighting system, giving each parameter an equal amount of weight.

### Identification of mega-environments

Mega-environments were identified for each individual year using three approaches: GGE biplot, AMMI biplot, and WAASB. The GE interaction analysis were carried out on individual year basis as the hybrids varied year to year. In GGE biplot, which-won-where biplot was generated by joining the outermost genotypes to form a polygon within which all other genotypes lay inside. A perpendicular was drawn to the sides of the polygon through the origin, and the environments were divided into different sectors, each with a different vertex genotype. Each sector containing an environment was considered a mega-environment, with vertex entry as a winning genotype. Winning genotype is defined by high grain yield and minimum GE interaction for the given mega environment.

For AMMI and WAASB methods, the genotype that had the highest nominal yield as determined by IPCA1 was the winner. The nominal yield was plotted against environmental IPCA1 scores.

The mega-environments were identified using the standard method [11]. A 10 x 10 environment matrix (frequency table) was constructed for each approach based on the classification of the mega environment by the three methods (GGE, AMMI, and WASSB). A score of one was assigned to all locations that occurred in the same environment. Locations occurring in distinct mega environments, however, received a score of zero. This procedure was applied to all three methods, and the results from the three matrices were combined to construct a combined matrix. A bias would emerge for locations that occurred in the majority of the years since hybrids were not tested at all ten locations for each year. To eliminate this bias, a final index was produced by dividing the scores from the combined matrix by the aggregate number of occasions each location pair occurred in a single mega environment across all the years. This final index for each location pair should be interpreted along with the number of occurrences for each year, which gives a picture of the association between the environments.

## Results

Between 2016 and 2019, the average grain yield was 7133 kg/ha across two agro-climatic zones (Zone 3 and 8) in Karnataka (Table 2). The mean grain yield in Zone-3 was 7122 kg/ha, while in Zone-8 it was 6954 kg/ha. During 2018, the highest mean grain yield of 8385 kg/ha was recorded. In contrast, 2016 had the lowest yield of 6093 kg/ha. The Arabhavi location in Zone 3 had the highest grain yield of 8916 kg/ha. Similarly, Dharwad in Zone-8 recorded the highest mean grain yield of 8711 kg/ha over four years.

### Individual and combined analysis

Individual analysis of variance for each location-year combination showed significant differences among the hybrids (p≤0.05), except for Bagalkot for the year 2018 (Table 3). The results reveal that the mean performance of test hybrids evaluated at each of the locations from 2016 to 2019 varied significantly (p≤ 0.05) from each other. Individual heritability in the year 2016 ranged from 62 to 93%, while combined heritability was 78%. In 2017, it ranged from 65 to 89%, while combined, it was 73%. Similarly, in 2018, it ranged from 31 to 85%, with a combined heritability of 79%, and in 2019, it ranged from 57 to 96%, with a combined heritability of 75%. Individual coefficients of variation ranged from 6.75 to 15.3 in 2016 while the combined value was 12.42; in 2017 it ranged from 9.44 to 18.8 while the combined value was 15.2; in 2018 it ranged from 6.78 to 18.4 while the combined value was 12.7; and in 2019 it ranged from 6.84 to 19.0 and the combined value was 14.08.

**Table 3:** Analysis of variance of individual location for trials 2016 to 2019.

2016					
Environment	MSS Genotypes	MSS Replication	MSS Error	Co-efficient of variation	Broad sense heritability
ARA	740**	1280**	151	13.5	0.80
BAI	207**	2.04	33.8	15.0	0.83
BEL	222**	65.1	43.6	13.8	0.80
DEV	195**	39.8	39.2	11.5	0.80
DWR	996**	463**	60.9	6.75	0.93
HAG	380**	238*	50.9	13.2	0.87
MUD	126*	325**	47.1	14.3	0.62
NIP	229*	108	86.3	13.5	0.62
SIR	42.4**	0.323	13.5	10.3	0.68
2017					
ARA	553**	220	170	15.1	0.69
BAG	405**	214	143	18.8	0.65
BAI	488**	39.3	128	16.8	0.74
DEV	251**	106	55.2	10.8	0.78
HAG	263**	25.7	30.1	9.44	0.89
MUD	405**	213	143	18.8	0.65
NIP	609**	105	78.5	19.5	0.87
SIR	351**	774**	120	11.6	0.66
2018					
ARA	297**	161	90.1	10.8	0.70
BAG	163	55.3	112	16.2	0.31
BAI	421**	45.2	50.5	7.93	0.88
BVG	290**	32.5	44.2	11.2	0.85
DWR	564**	35.1	84.1	13.2	0.85
MUD	242*	19.8	78.5	12.4	0.68
NIP	1508**	339	394	18.4	0.74
SIR	1478**	123	68.9	6.78	0.95
2019					
ARA	535**	138	135	12.8	0.75
BAG	191*	36.8	71.5	12.9	0.63
BAI	240**	55.9	25.3	11.9	0.90
BEV	834*	536	284	19.0	0.66
DWR	303*	143	131	15.0	0.57
HAG	447**	879**	15.3	6.84	0.97
MUD	488**	28	89.4	11.7	0.81
SIR	113*	132*	48.9	10.5	0.60

* significance at p ≤ 0.05;

** significance at p ≤ 0.05.

A combined analysis of variance for each year indicated significant genotype, environment, and GE interactions (Table 4). Among the sources of variation, the environmental factor recorded the highest sum of squares, indicating the highest variation to the extent of 85.0%, 62.04%, 69.32% and 66.40% respectively, across all four years. The proportion of GE interaction accounted for 9.50%, 24.68%, 18.36% and 21.53% respectively. From 2016 to 2019, genotypic variation accounted for 5.50%, 13.28%, 12.32% and 12.07% of the total variation, Across all the years, GE interaction recorded a greater proportion of variation than genotype, indicating its significance for hybrid performance in various environments. The portion of variation useful for mega-environment classification is both genotype sum of squares (GSS) and GE sum of squares (GE SS), which together account for 15.0%, 37.95%, 30.67% and 33.60% of total variation, respectively. The GE SS is composed of both noise and signal SS, with noise SS producing spurious results as a result of uncontrolled experimental variations within experimental fields [29]. Noise SS is calculated by multiplying GE df by GESS, which is 23.54%, 35.10%, 27.17%, and 34.71% from 2016 to 2019, respectively, whereas signal SS is calculated by subtracting noise SS from the GESS, which is 76.46%, 64.90%, 72.83%, and 65.29% from 2016 to 2019. The relevant variation of the total treatment SS (genotype SS + signal SS) after discounting noise SS is only 13.05%, 32.07%, 27.04% and 28.23% for the years 2016 to 2019, respectively.

**Table 4:** Combined analysis of variance for trials from year 2016 to 2019.

	2016			2017			2018			2019		
Source	df	Mean Sum of Squares	Proportion of variation	df	Mean Sum of Squares	Proportion of variation	df	Mean Sum of Squares	Proportion of variation	df	Mean Sum of Squares	Proportion of variation
Environment	8	31126.8**	85.00	7	10874.26**	62.04	7	17617.05**	69.32	7	9784.49**	66.40
Replication (Env)	18	280.08**		16	212.24*		16	101.41		16	243.53**	
Genotype	14	1150.93**	5.50	14	1160**	13.28	11	1992.04**	12.32	11	1131.82**	12.07
GE Interaction	112	248.4**	9.50	98	309**	24.68	77	424.31**	18.36	77	288.35**	21.53
Residuals	252	58.48		224	108		176	115.34		176	100.11	
CV (%)	12.42			15.20			12.76			14.08		
Overall mean	61.55			68.53			84.17			71.06		

** significance at p ≤ 0.01.

### AMMI and GGE biplot analysis

**Fig 2:**
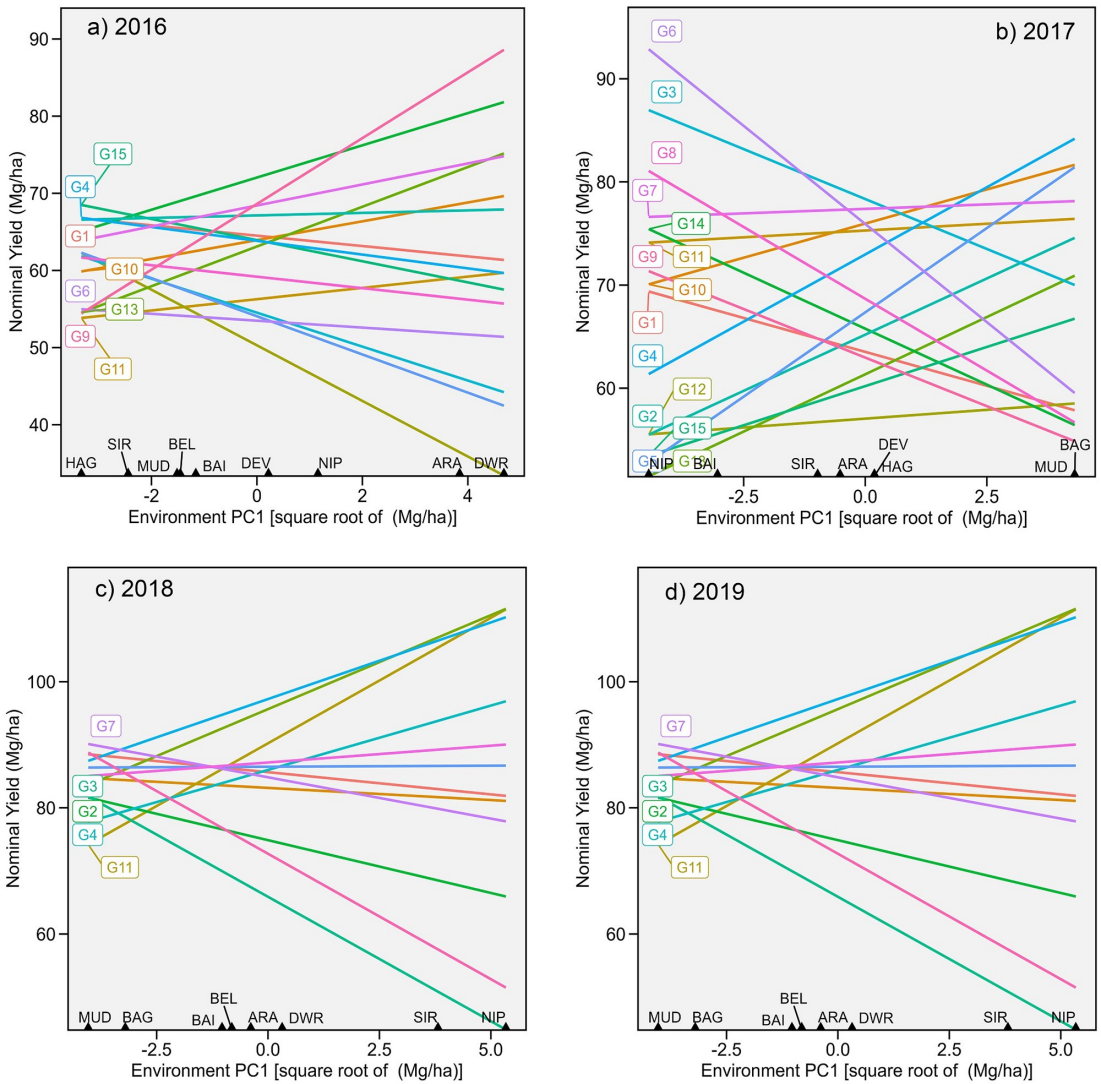
Nominal yield (IPCA-1) plotted against Environmental IPCA-1 scores from AMMI analysis.

The results of the AMMI analysis for all four years are presented in Table 5. The first two IPCs (interaction principal components) obtained by singular value decomposition of GE interactions were statistically significant (*p*< 0.01) by the *F* test. IPC1 accounted for 40.00%, 45.00%, 47.00%, and 34.10% of the total GESS variation for the years 2016 to 2019, respectively, but signal SS is lower than GESS, so IPC1 accounted for 52.31%, 69.37%, 64.59%, and 52.27% of the total signal SS. A plot of nominal yield against environmental scores is presented in Fig 2. The genotype with the highest nominal yield is considered the winning genotype for the given location.

**Table 5:** AMMI analysis of the trials from 2016 to 2019.

Source of variation	2016			2017			2018			2019		
	df	Mean sum of squares	Proportion (%)	df	Mean sum of squares	Proportion (%)	df	Mean sum of squares	Proportion (%)	df	Mean sum of squares	Proportion (%)
Environment	8	31127**		7	10874.26**		7	17617**		7	9784**	
Replication (Env)	18	280.1**		16	212.24*		16	101.4		16	243.5**	
Genotypes	14	1151**		14	1163.59**		11	1992**		11	1132**	
Genotype x Environment	112	248.4**		98	308.97**		77	424.3**		77	288.4**	
PC1	21	529.8**	40.00	20	681.55**	45.00	17	904**	47.00	17	445.7**	34.10
PC2	19	397.6**	27.20	18	457.22**	27.20	15	480.4**	22.10	15	489.2**	33.10
Residuals	252	58.48		224	108.45		176	115.3		176	100.1	
Total	404	774.1		457	395.31		287	696.3		287	434.4	

** significance at p ≤ 0.01.

**Fig 3:**
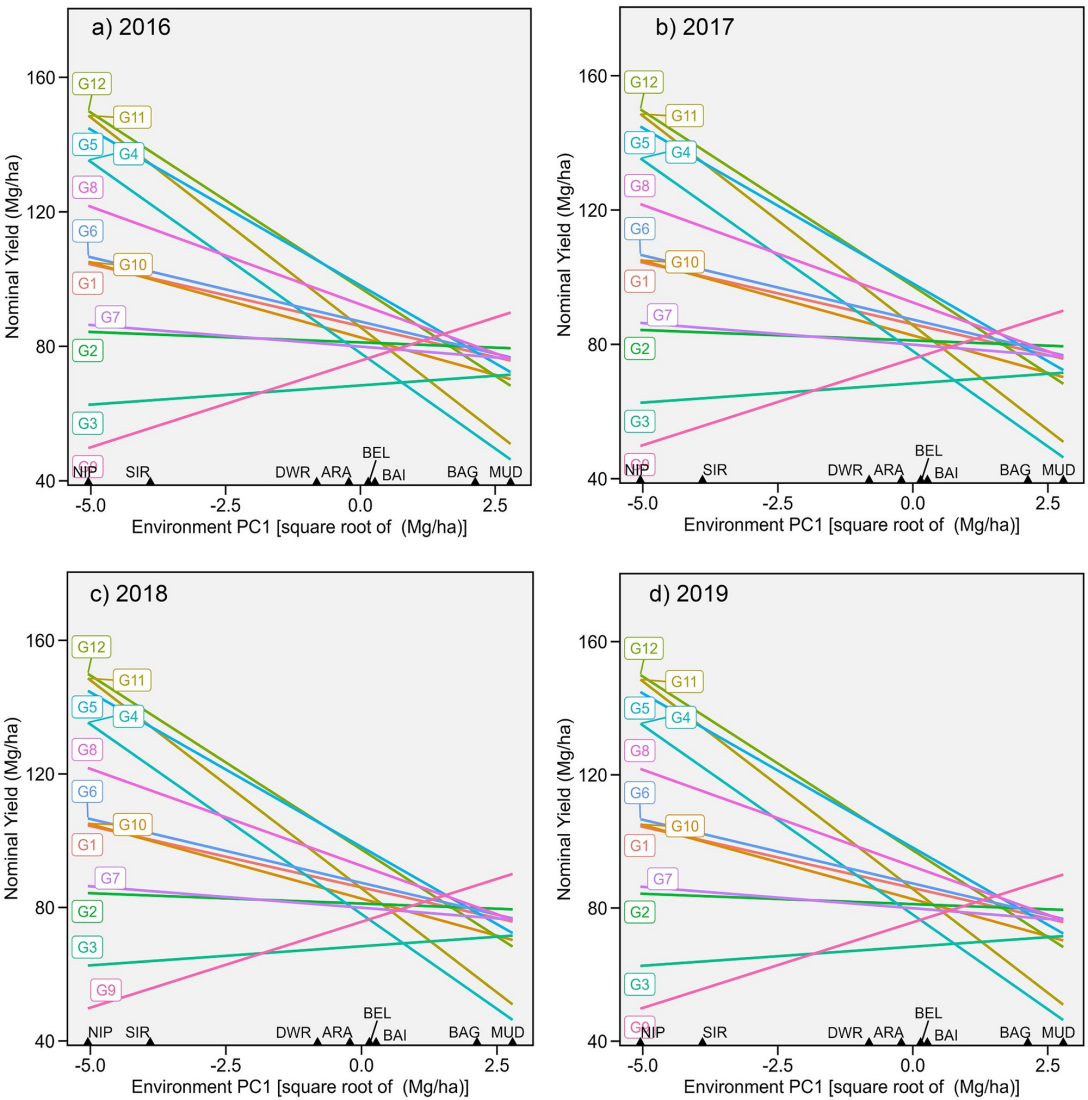
Nominal yield (IPCA-1) plotted against Environmental IPCA-1 scores from WAASY analysis.

WAASB is a combination of BLUP and AMMI methods, in which the BLUP of GE interaction is subjected to AMMI analysis. The IPC1 using the WAASB method accounted for 40.47%, 43.46%, 48.20%, and 33.74% of the total GESS variation for the years 2016 to 2019. Similar to AMMI analysis, winning genotypes for each location were identified by plotting nominal yield against environmental scores (Fig 3).

**Fig 4:**
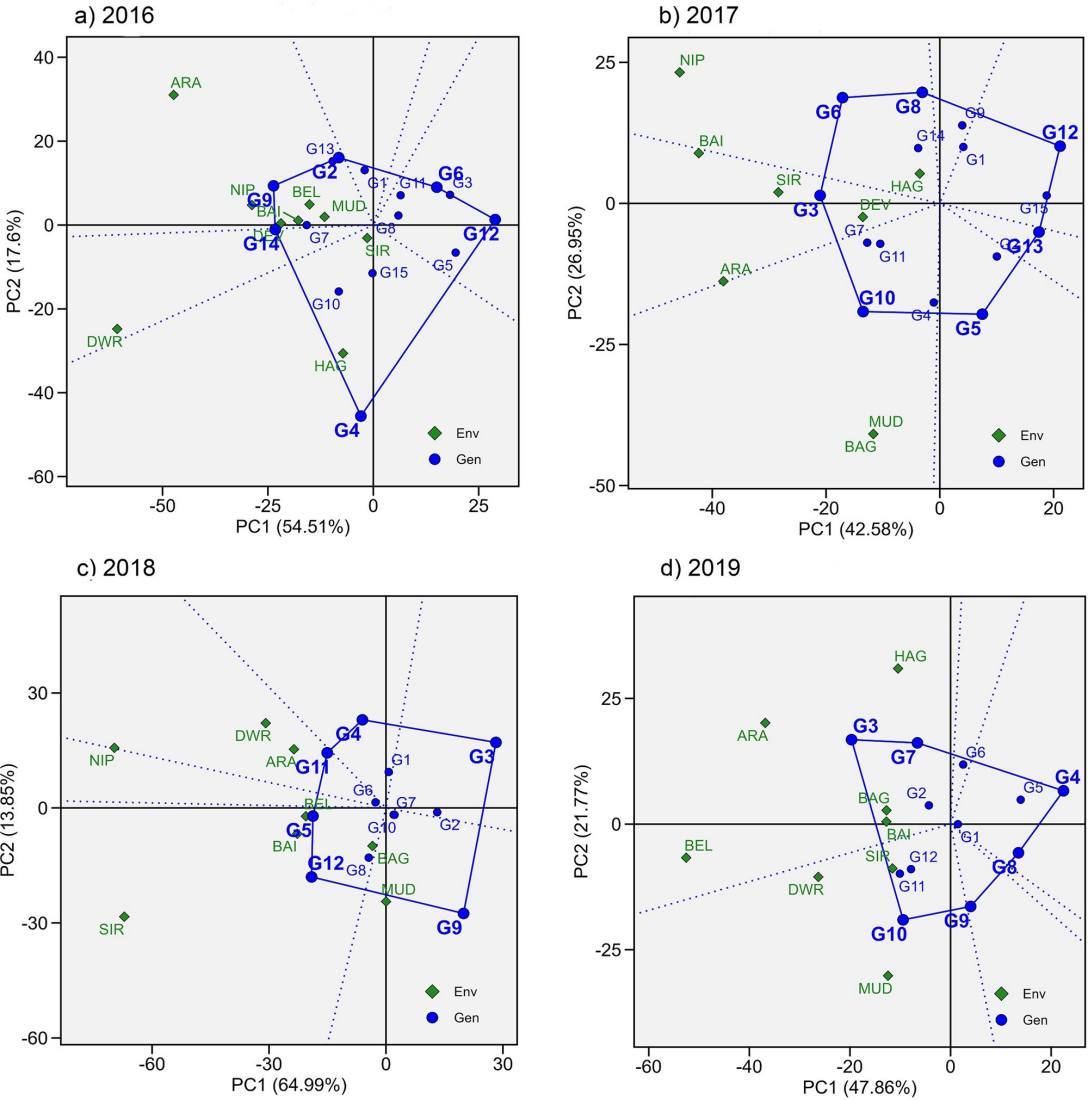
Which-won-where view of the GGE biplot for trials from 2016 to 2019. Note: Scaling = 0, Centering = 2, SVP = 2.

In GGE biplot analysis, the first two PC (principal components) explained 72.11%, 69.53%, 78.84%, and 69.63% of the total genotype and GE variation for the years 2016 to 2019, respectively. The winning genotypes for each of the locations were identified using the which-won-where view of the biplot produced as per [29] (Fig 4).

### Stability indices

For the year 2016, the AMMI 1 biplot identified genotypes G2, G1, and G4 as stable hybrids. Whereas, for the year 2017, G11, G7, and G10 were identified as stable; in 2018, hybrids G6, G8, and G10 were recognized as stable; and in 2019, G11, G12, and G2 were observed as stable (Table 6). The AMMI 2 biplot is represented by two principal components, IPC 1 and IPC 2, as ‘x’ and ‘y’ axes, respectively. The genotypes closer to origin indicate low GE interaction. The genotypes that are closer to the location emphasize adaptation to that environment. Hybrids G8 and G11 for the year 2016 displayed the least GE interaction, whereas hybrids G11 and G7 for the year 2017, G10, G6, and G2 for the year 2019 showed the least GE interaction.

**Table 6:** Mega environment identification through AMMI, WAASB and GGE method for year 2016 to 2019.

Methodology	Year	Mega environment	Winning genotype	Environment
GGE	2016	I	G4	HAG, SIR
		II	G14	DWR
		III	G9	DEV, MUD, BAI, BEL, NIP, ARA
	2017	I	G10	ARA, BAG, MUD DWR
		II	G3	BAI, SIR, DEV
		III	G6	NIP, HAG
	2018	I	G11	DWR, ARA, NIP
		II	G12	BAI, SIR, BAG
		III	G9	MUD
	2019	I	G3	ARA, HAG, BEV, BAG, BAI
		II	G10	DWR, SIR, MUD
AMMI	2016	I	G9	ARA, DWR
		II	G14	DEV, NIP, BAI, BEL, MUD
		III	G15	HAG, SIR
	2017	I	G6	NIP, BAI
		II	G3	SIR, ARA, DEV, HAG
		III	G4	MUD, BAG
	2018	I	G12	SIR, NIP
		II	G5	BAG, BAI, BEL, ARA, DWR
		III	G7	MUD
	2019	I	G10	BAI, DWR, SIR, MUD
		II	G3	BEV, HAG, ARA
WAASB	2016	I	G9	ARA, DWR
		II	G4	NIP, DEV, BAI, BEL, MUD
		III	G15	HAG, SIR
	2017	I	G4	MUD, BAG
		II	G3	HAG, DEV, ARA, SIR
		III	G6	NIP, BAI
	2018	I	G12	NIP, SIR, DWR
		II	G5	ARA, BEL, BAI
		III	G9	BAG, MUD
	2019	I	G3	BEL, ARA
		II	G10	DWR, HAG, SIR, BAG, BAI, MUD

**Fig 5:**
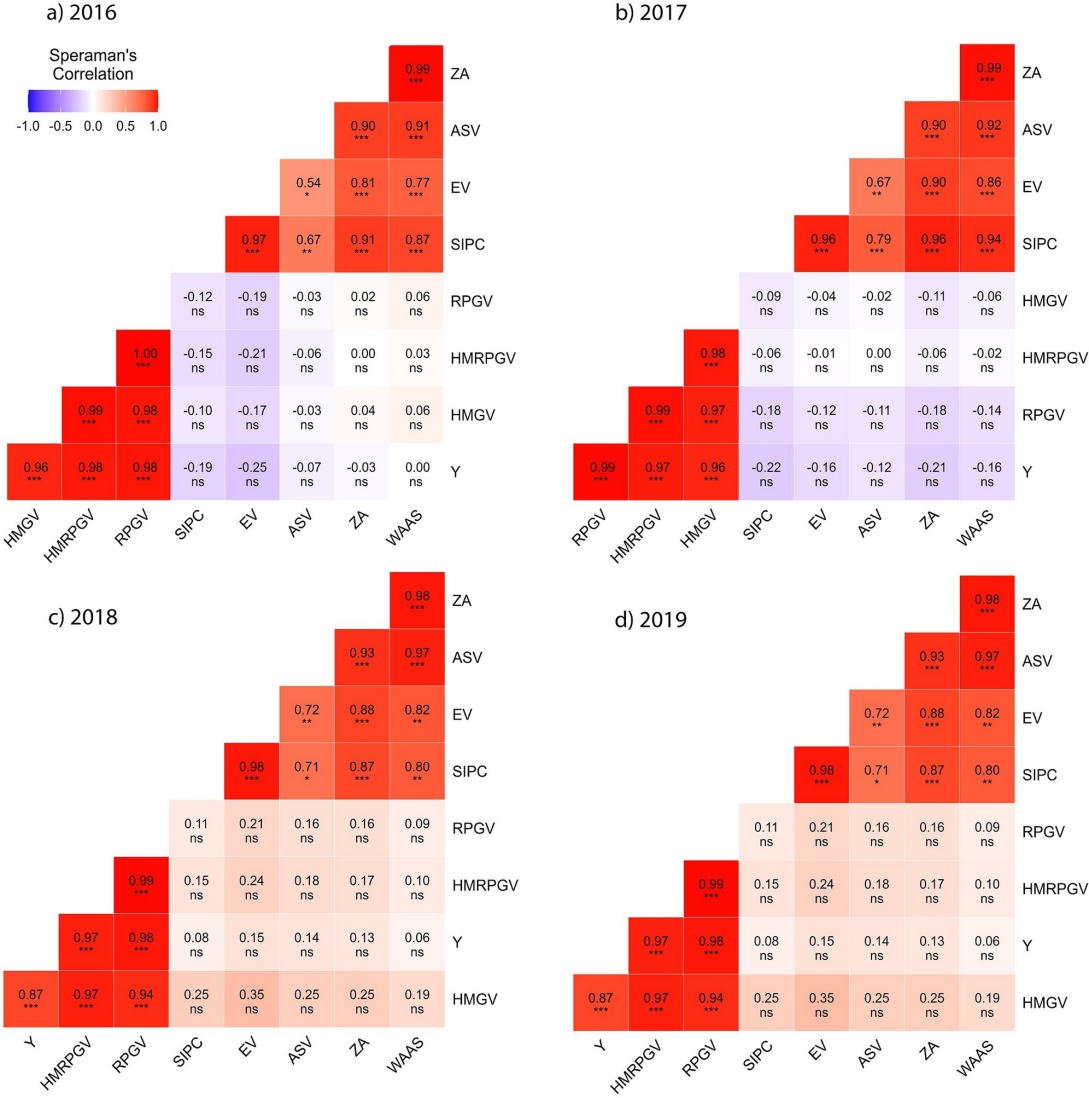
Heatmap showing correlation between AMMI and BLUP stability indices along with grain yield. Note: ns–p > = 0.05; *—p < 0.05; **—p < 0.01; ***—p < 0.0001.

Based on the ranking of stability indices (S3 and S4 Tables), a heat map was produced between various stability indices of AMMI, BLUP, and WASSY (Fig 5). The rank correlation among stability indices revealed that the BLUP and AMMI indices formed two distinct clusters. A high correlation was observed within the clusters, whereas a low correlation was observed between the clusters. From 2016 to 2019, the correlation between AMMI indices and grain yield ranged from -0.25 to 0, -0.22 to -0.12, 0.06 to 0.15, and -0.52 to -0.36, respectively, whereas the correlation between BLUP indices and grain yield ranged from 0.67* to 0.97*, 0.96** to 0.99**, 0.87** to 0.98**, and 0.99** to 1.00**. Thus, in general, the BLUP and AMMI indices had a high and low/negative correlation with grain yield, respectively.

The WAASY index showed significant correlation with both the AMMI and BLUP indices. Thus, it shows that the WAASY index considers both grain yield and GE interaction when identifying the best hybrids. The correlation of WAASY index with AMMI indices for the years 2016 to 2017 ranged from 0.40 to 0.75**, 0.36 to 0.46, 0.79** to 0.85**, and 0.20 to 0.34 respectively, whereas that with BLUP indices ranged from 0.64* to 0.68**, 0.76** to 0.82**, 0.49 to 0.59*, and 0.66* to 0.67* respectively. WAASB stability indices and simultaneous selection have been used to identify stable and early-maturity genotypes in soybean [22] where WAASB identified G17 and G11 as stable genotypes. A 65/35 weightage was followed to estimate WAASBY index in which G18 and G 31 were identified as stable genotypes.

### Mega environment analysis

All three methods, GGE, AMMI, and WAASB, identified the same number of mega-environments for each year, namely three mega-environments from 2016 to 2018, and two mega-environments from 2019 to 2019 (Table 6).

A two-way table was developed, and if two locations were in the same mega environment, they were given a score of one. For a given year, the highest score a pair of environments can have is three (if both locations fall under the same mega environment using all three methods), and the lowest score is zero (both locations not occurring once using all three methods). Furthermore, the scores for each location pair were pooled to obtain observed scores, which were then divided by expected scores (considering all locations were to be grouped in a single mega environment) to obtain a pooled index. The two-way table representing the occurrence of locations in a single mega environment through methods GGE, AMMI, and WAASB is presented in Tables 7–9, respectively. A two-way table with a pooled index is presented in Table 10.

**Table 7:** Frequency of each location pair in the same mega environment as identified by GGE biplot analysis.

	Arabhavi	Bagalkot	Belavatagi	Hagari	Mudhol	Siruguppa	Bailhongal	Devihosur	Dharwad	Nippani
Arabhavi	0									
Bagalkot	2	0								
Belavatagi	2	1	0							
Hagari	1	1	1	0						
Mudhol	2	1	1	0	0					
Siruguppa	0	1	0	1	1	0				
Bailhongal	2	2	2	1	0	2	0			
Devihosur	1	0	1	0	1	0	2	0		
Dharwad	1	0	0	0	1	1	0	0	0	
Nippani	1	0	1	0	1	0	1	1	1	0

**Table 8:** Frequency of each location pair in the same mega environment as identified by AMMI biplot analysis.

	Arabhavi	Bagalkot	Belavatagi	Hagari	Mudhol	Siruguppa	Bailhongal	Devihosur	Dharwad	Nippani
Arabhavi	0									
Bagalkot	1	0								
Belavatagi	2	1	0							
Hagari	1	0	1	0						
Mudhol	0	1	1	0	0					
Siruguppa	1	0	0	1	1	0				
Bailhongal	1	1	2	0	2	1	0			
Devihosur	0	0	1	1	1	0	1	0		
Dharwad	2	1	1	0	1	1	2	0	0	
Nippani	0	0	1	0	1	1	2	1	0	0

**Table 9:** Frequency of each location pair in the same mega environment as identified by WAASB biplot analysis.

	Arabhavi	Bagalkot	Belavatagi	Hagari	Mudhol	Siruguppa	Bailhongal	Devihosur	Dharwad	Nippani
Arabhavi	0									
Bagalkot	0	0								
Belavatagi	2	0	0							
Hagari	1	1	0	0						
Mudhol	0	3	1	1	0					
Siruguppa	1	1	0	3	1	0				
Bailhongal	1	1	2	1	2	1	0			
Devihosur	1	0	1	1	1	1	1	0		
Dharwad	1	1	0	1	1	2	1	0	0	
Nippani	0	0	1	0	1	1	2	1	1	0

**Table 10:** Pooled index along with sum of scores from all three methods (in parenthesis) for each location pair.

	Arabhavi	Bagalkot	Belavatagi	Hagari	Mudhol	Siruguppa	Bailhongal	Devihosur	Dharwad	Nippani
Arabhavi	0									
Bagalkot	0.33 (3)	0								
Belavatagi	0.67 (3)	0.33 (2)	0							
Hagari	0.44 (3)	0.33 (2)	0.33 (2)	0						
Mudhol	0.17 (4)	0.56 (3)	0.33 (3)	0.11 (3)	0					
Siruguppa	0.17 (4)	0.22 (3)	0.00 (3)	0.67 (3)	0.25 (4)	0				
Bailhongal	0.33 (4)	0.44 (3)	0.67 (3)	0.22 (3)	0.33 (4)	0.33 (4)	0			
Devihosur	0.50 (2)	0.00 (1)	1.00 (1)	0.33 (2)	0.50 (2)	0.33 (2)	0.67 (2)	0		
Dharwad	0.44 (3)	0.33 (2)	0.11 (3)	0.17 (2)	0.33 (3)	0.44 (3)	0.33 (3)	0.00 (1)	0	
Nippani	0.11 (3)	0.00 (2)	0.50 (2)	0.00 (2)	0.33 (3)	0.22 (3)	0.56 (3)	0.50 (2)	0.33 (2)	0

All three methods grouped location pairs Belavatagi-Mudhol, Belavatagi-Bailhongal, Belavatagi-Devihosur, Belavatagi-Nippani, Hagari-Siruguppa, Mudhol-Devihosur, Mudhol-Nippani, and Nippani-Devihosur under the same mega environment in 2016. In 2017, only Bagalkot-Mudhol was grouped under the same mega environment. In 2018, none of the location pairs were grouped under a single mega environment, which could indicate a unique interaction among the locations such that any of the location pairs could not be placed in a single mega environment using all three methods. Similarly, Arabhavi-Belavatagi, Mudhol-Siruguppa, Mudhol-Dharwad, and Siruguppa-Dharwad all occurred together in a single mega environment in 2019.

Belavatagi-Devihosur recorded the highest value of one, but this occurred only once during the MLT trials. Location pairs Arabhavi-Belavatagi, Belavatagi-Bailhongal, and Hagari-Siruguppa each had three occurrences and displayed a value of 0.67. Bailhongal-Devihosur, on the other hand, had a value of 0.67 with two occurrences. Similarly, location pairs Bagalkot-Mudhol and Bailhongal-Nippani showed three occurrences with a value of 0.56. On the contrary, location pairs Belavatagi-Siruguppa with three occurrences, Bagalkot-Nippani, Hagari-Nippani with two occurrences, and Devihosur-Bagalkot and Devihosur-Dharwad with one occurrence each had zero value. Dharwad showed a low value with all the locations, *i*.*e*., less than 0.5.

## Discussion

The hybrids with minor GE interactions could be identified using AMMI stability indices. The association between BLUP indices and grain yield makes it evident that BLUP indices identified the hybrids with high grain yield. BLUP is opined to be the best alternative to AMMI when the data set is large; however, the advantage of AMMI is that it can be used for modelling and knowing interactions apart from imputing the missing values [34].

WAASBY which weighs both grain yield and GE interaction identified the best stable hybrids with respect to both the parameters. Hybrids G14, G2, and G7 for 2016, G7, G11 and G3 for 2017, G5, G6 and G10 for 2018, and G2, G10 and G1 for 2019 were identified as the best-performing hybrids. In a similar study WAASBY index was used to identify, stable maize hybrids 34], cotton genotypes for salt effected soils [35], maize in Huanghuaihai region of China [36] and soyabean genotypes resistant to anthracnose [37].

The findings from the current study are in accordance with the results obtained in cotton [35], and maize [36]. The proportion of the total useful variation available for mega-environment classification ranged between 13% and 32%. A similar finding revealed that the relevant information was typically 10% to 40% of the treatment variation [10]. The classification of mega environments is useful for identifying similar-performing environments and grouping them for the release of best-yielding cultivars. Because hybrid performance varies from location to location and year to year in a multi-location trial, poor-performing hybrids can be eliminated and new hybrids can be incorporated [23]. Because the same set of cultivars is not evaluated every year, mega-environments were identified for each year in the current study using three methods: AMMI, GGE, and WAASB.

The relationship between the locations was discovered by aggregating the mega-environment pattern scores for each individual year. The location pairs Arabhavi-Belavatagi and Bailhongal-Belavatagi showed the highest value of 0.67 (3), indicating that these pairs shared a common winning genotype in two of the three trials conducted. By transitive property, Arabhavi-Bailhongal should also share the same winning genotype, but interestingly, Arabhavi-Bailhongal showed a low value of 0.33 (4), suggesting that they shared the winning genotype only in one of the three trials. Similarly, the most discriminating and representative environments as well as ideal genotypes were identified in barley [38], oat [39], finger millet [40], and maize [41], which support the results obtained in the present study.

The relationship between locations Belavatagi and Bailhongal is intriguing because they belong to distinct agro-climatic zones, Zone-3 and 8 respectively, but in spite of that, they shared a common winning genotype on the majority of occasions (0.67). Arabhavi, Bailhongal, and Belavatagi, on the other hand, cannot be classified under a single mega environment. This pattern implies a complex interrelationship across environments, making the formation of mega-environments a challenging task. In contrast, the location pair Hagari-Siruguppa in Zone-3 recorded a value of 0.67 (3) and neither location had a strong correlation with any of the other locations. As a result, these two locations can be considered to constitute a mega-environment of their own. Similarly, the Dharwad location didn’t show a value greater than 0.5 with any of the locations. Hence, Dharwad can be considered a distinct location from the rest of the others.

The location pairs Arabhavi-Nippani (different agro climatic zones (AGZ)), Dharwad-Hagari (different AGZ), and Hagari-Mudhol (same AGZ) showed a value of 0.11 (3). Whereas Arabhavi-Mudhol (same AGZ) and Arabhavi-Siruguppa (same AGZ) recorded a value of 0.17 (3), Siruguppa-Bagalkot (same AGZ) and Nippani and Siruguppa (different AGZ) showed a value of 0.22 (3). The low value indicates that these location pairs rarely share a winning genotype in common, implying that they are more likely to be located in separate mega-environments. Locations within the same AGZ as well as locations belonging to distinct agro-climatic zones exhibited low values, highlighting that all locations within a single AGZ do not necessarily share a similar winning genotype. As a result, recommending hybrids based on an AGZ may not be the most effective strategy.

One of the core ideas of the mega environment is to group various growing regions into a single, homogeneous cluster, but the present study revealed that this cannot be done simply, especially when the cultivars vary annually. The agro-climatic zones (AGZ) are categorised based on environmental factors such as rainfall, temperature, soil type, etc. but do not take into consideration GE interaction, rendering using only AGZ for cultivar release less effective. The current analysis describes the likelihood of each location pair occurring in the same or different mega-environment. To categorise sites into distinct mega-environments, further trials are necessary.

## Conclusion

GGE, AMMI, and WAASB methods were adopted to analyse the mega-environments for the specific years 2016, 2017, 2018, and 2019. AMMI and BLUP indices are inclined to identify hybrids with low GE interaction and high grain yield, respectively. In contrast, WAASBY identified hybrids that performed well for both parameters. Based on the mega-environment pattern for each individual year, the probability of each location pair occurring in the same or different mega-environment was estimated. Location pairs such as Arabhavi-Belavatagi and Bailhongal-Belavatagi often shared the same winning genotype, making it more probable that they would occur in the same mega-environment. On the other hand, Arabhavi-Nippani, Hagari-Mudhol, and Dharwad-Hagari, which rarely shared the same winning genotype, can be considered to form independent mega-environments. The lack of a distinct mega-environment pattern between the locations suggests complicated GE interactions. Therefore, it is important to emphasize breeding for widely adaptable cultivars to ensure that performance is consistent across environments.

## Supporting information

S1 Table Hybrids evaluated at different locations form year 2016 to 2019. (PDF)

S2 Table BLUP (Best Linear Unbiased Predictor) values of hybrids for year 2016 to 2019. (PDF)

S3 Table Ranking of genotypes based on HMGV, RPGV, HMRPGV (BLUP based stability indices). (PDF)

S4 Table Ranking of hybrids based on AMMI and WAASB stability indices. (PDF)

S1 Appendix R code for analysis of individual ANOVA and combined ANOVA. (PDF)

S2 Appendix R code for estimation of stability indices. (PDF)
