# Supplementary material for: Importance of mega-environments in evaluation and identification of climate resilient maize hybrids (Zea mays L.)
Source: PLoS One. 2023 Dec 14;18(12):e0295518. doi: 10.1371/journal.pone.0295518 (PMC10721017; doi:10.1371/journal.pone.0295518)
Supplement: S1 Table — (PDF) [file pone.0295518.s001.pdf]

S1 Table. Hybrids evaluated at different locations form year 2016 to 2019.

2016				2017				2018				2019			
Hybrid	Hybrid Code	Environment	Environment Code	Hybrid	Hybrid Code	Environment	Environment Code	Hybrid	Hybrid Code	Environment	Environment Code	Hybrid	Hybrid Code	Environment	Environment Code
BRMH-10	G1	Arabhavi	ARA	RCRMH 4-1	G1	Arabhavi	ARA	AH-8087	G1	Arabhavi	ARA	AH-8245R	G1	Arabhavi	ARA
GH-1427	G2	Belavatagi	BEL	GH-1427	G2	Bagalkot	BAG	GPMH-1101 (LC)	G2	Bagalkot	BAG	RCRMH-2	G2	Bagalkot	BAG
DMH-100-21	G3	Hagari	HAG	NK-6240 (NC)	G3	Hagari	HAG	GH-1314	G3	Belavatagi	BEL	900 M Gold (PC)	G3	Belavatagi	BEL
CAH-1526	G4	Mudhol	MUD	GH-1501414	G4	Mudhol	MUD	RCRMH-3	G4	Mudhol	MUD	GH-0727 (LC)	G4	Hagari	HAG
GH-1436	G5	Siruguppa	SIR	BRMH-643	G5	Siruguppa	SIR	900 M Gold (PC)	G5	Siruguppa	SIR	GH-1765	G5	Mudhol	MUD
GH-0727 (LC)	G6	Bailhongal	BAI	900 M Gold (PC)	G6	Bailhongal	BAI	RCRMH-2 (Check)	G6	Bailhongal	BAI	GH-16532	G6	Siruguppa	SIR
BIOSEED (NC)	G7	Devihosur	DEV	GH-160224	G7	Nippani	NIP	AH-8070	G7	Dharwad	DWR	AH-4158	G7	Bailhongal	BAI
CAH-1454	G8	Dharwad	DWR	AH-8071R	G8			GH-17180	G8	Nippani	NIP	GPMH-1101 (LC)	G8	Dharwad	DWR
GH-150145	G9	Nippani	NIP	GPMH-1101 (LC)	G9			GH-0727 (LC)	G9			RCRMH-9	G9		
900 M Gold (PC)	G10			RCRMH-3	G10			RCRMH-4	G10			NK-6240 (NC)	G10		
BRMH-1	G11			GH-1501525	G11			NK-6240 (NC)	G11			AH-8183	G11		
DMH-100-1	G12			GH-0727	G12			AH-4158	G12			GH-1720	G12		
AH-7005	G13			AH-7188	G13										
GH-150225	G14			GH-160295	G14										
AH-7118	G15			BRMH-556	G15										

Note: LC – Local Check, NC – National Check, PC- Private Check. Refer Table 2 to know the names of environment code.
