# Supplementary material for: Importance of mega-environments in evaluation and identification of climate resilient maize hybrids (Zea mays L.)
Source: PLoS One. 2023 Dec 14;18(12):e0295518. doi: 10.1371/journal.pone.0295518 (PMC10721017; doi:10.1371/journal.pone.0295518)
Supplement: S2 Table — (PDF) [file pone.0295518.s002.pdf]

S2 Table. BLUP (Best Linear Unbiased Predictor) values of hybrids for year 2016 to 2019.

2016			2017			2018			2019		
GEN	GY	BLUPg	Gen	GY	BLUPg	GEN	GY	BLUPg	GEN	GY	BLUPg
G14	7204	8.22	G7	8106	6.89	G5	9725	10.30	G3	8124	7.59
G9	6820	5.54	G3	8010	6.18	G12	9565	9.04	G10	7987	6.57
G7	6820	5.38	G10	7927	5.57	G11	9024	4.78	G7	7556	3.36
G2	6730	4.37	G11	7725	4.10	G8	8719	2.38	G2	7469	2.71
G1	6400	2.31	G6	7714	4.01	G6	8652	1.85	G12	7425	2.38
G15	6330	1.86	G4	7586	3.08	G4	8602	1.45	G11	7416	2.31
G10	6320	1.86	G8	7150	-0.11	G1	8566	1.17	G6	7130	0.19
G4	6340	1.79	G5	7045	-0.87	G7	8485	0.53	G9	6852	-1.89
G13	6300	1.21	G14	6972	-1.41	G10	8316	-0.79	G1	6718	-2.89
G8	5919	-1.86	G2	6797	-2.69	G2	7487	-7.32	G8	6449	-4.89
G11	5627	-4.14	G9	6779	-2.82	G9	7272	-9.01	G5	6231	-6.52
G3	5452	-5.51	G1	6776	-2.84	G3	6590	-14.38	G4	5910	-8.91
G5	5406	-5.88	G13	6381	-5.73						
G6	5348	-6.33	G15	6340	-6.03						
G12	5029	-8.83	G12	6161	-7.34						

Note: BLUPg – Best linear unbiased predictor of genotypes GY: Grain yield (kg/ha)
