# Supplementary material for: Importance of mega-environments in evaluation and identification of climate resilient maize hybrids (Zea mays L.)
Source: PLoS One. 2023 Dec 14;18(12):e0295518. doi: 10.1371/journal.pone.0295518 (PMC10721017; doi:10.1371/journal.pone.0295518)
Supplement: S3 Table — (PDF) [file pone.0295518.s003.pdf]

S3 Table. Ranking of genotypes based on HMGV, RPGV, HMRPGV (BLUP based stability indices).

2016									
GEN	GY	HMGV	HMGV_R	RPGV	RPGV_Y	RPGV_R	HMRPGV	HMRPGV_Y	HMRPGV_R
G1	6450	57.5	4	1.07	65.6	5	1.05	64.8	5
G10	6390	53.5	8	1.02	62.8	8	1.01	62.1	8
G11	5630	47.9	13	0.904	55.6	11	0.896	55.1	12
G12	5030	45.5	15	0.839	51.6	15	0.834	51.4	15
G13	6310	51.3	10	0.997	61.4	9	0.976	60.1	9
G14	7200	62.4	1	1.16	71.5	1	1.16	71.3	1
G15	6390	55.9	6	1.04	64.2	6	1.04	63.7	6
G2	6710	60	2	1.1	67.9	2	1.1	67.5	2
G3	5450	48.9	11	0.902	55.5	12	0.899	55.4	11
G4	6380	55.8	7	1.04	64.1	7	1.02	63	7
G5	5410	48.7	12	0.897	55.2	13	0.892	54.9	13
G6	5350	46	14	0.867	53.4	14	0.86	52.9	14
G7	6840	58.9	3	1.1	67.9	3	1.09	67.3	3
G8	5920	52.6	9	0.97	59.7	10	0.968	59.6	10
G9	6860	56.9	5	1.09	67	4	1.07	65.8	4
**2017**									
GEN	GY	HMGV	HMGV_R	RPGV	RPGV_Y	RPGV_R	HMRPGV	HMRPGV_Y	HMRPGV_R
G1	6350	60.7	12	0.931	63.8	11	0.928	63.6	11
G10	7600	71	5	1.1	75.2	4	1.09	74.4	5
G11	7530	72.2	4	1.1	75.2	5	1.09	75	3
G12	5700	54	15	0.842	57.7	15	0.832	57	15
G13	6130	57.7	13	0.9	61.7	13	0.89	61	13
G14	6580	61.2	9	0.955	65.4	10	0.941	64.5	10
G15	6020	57.2	14	0.886	60.7	14	0.879	60.2	14
G2	6520	62.3	8	0.957	65.6	9	0.952	65.2	8
G3	7830	74.3	1	1.14	78	1	1.13	77.1	1
G4	7300	68.5	6	1.06	72.4	6	1.05	71.9	6
G5	6740	60.9	11	0.973	66.7	8	0.951	65.2	9
G6	7590	72.6	3	1.11	76.3	3	1.09	74.8	4
G7	7740	73.5	2	1.12	76.7	2	1.12	76.7	2
G8	6870	65.3	7	1	68.8	7	0.992	67.9	7
G9	6300	60.9	10	0.931	63.8	12	0.925	63.4	12

Continued…

					2018				
GEN	GY	HMGV	HMGV_R	RPGV	RPGV_Y	RPGV_R	HMRPGV	HMRPGV_Y	HMRPGV_R
G1	8570	82.2	3	1.03	86.5	5	1.02	86.1	5
G10	8320	79.2	8	0.993	83.5	9	0.991	83.4	9
G11	9020	82.1	4	1.06	89.2	3	1.04	87.6	3
G12	9570	87.8	2	1.12	94	2	1.11	93.6	2
G2	7490	71.5	10	0.899	75.7	10	0.895	75.3	10
G3	6590	64.4	12	0.807	67.9	12	0.8	67.3	12
G4	8600	78.7	9	1.01	85.2	7	1	84.1	8
G5	9730	90.3	1	1.14	96	1	1.14	95.9	1
G6	8650	80.2	7	1.02	86.1	6	1.02	85.4	6
G7	8480	80.4	6	1.01	85.2	8	1.01	84.8	7
G8	8720	81.8	5	1.03	87	4	1.03	86.5	4
G9	7270	68.4	11	0.875	73.7	11	0.856	72	11
					2019				
GEN	GY	HMGV	HMGV_R	RPGV	RPGV_Y	RPGV_R	HMRPGV	HMRPGV_Y	HMRPGV_R
G1	6720	62.8	9	0.944	67.1	9	0.94	66.8	9
G10	7990	75	2	1.12	79.4	2	1.11	78.9	2
G11	7420	64.7	7	1.02	72.2	6	0.998	70.9	7
G12	7430	69	5	1.04	73.8	5	1.03	73.2	5
G2	7470	71.4	4	1.06	75.1	4	1.05	74.8	4
G3	8120	75.5	1	1.13	80.3	1	1.12	79.8	1
G4	5910	57.1	12	0.852	60.5	12	0.847	60.2	12
G5	6230	58.6	11	0.884	62.8	11	0.88	62.6	11
G6	7130	67.4	6	1.01	71.6	7	1	71.2	6
G7	7560	72.2	3	1.07	75.9	3	1.06	75.4	3
G8	6450	61.4	10	0.917	65.2	10	0.914	65	10
G9	6850	64.1	8	0.969	68.9	8	0.954	67.8	8

Note: GY - Grain yield (kg/ha), R – Rank of the given parameter, HMGV - Harmonic mean of genotypic values, RPGV - Relative performance of the genotypic values, HMRPGV - harmonic mean of the relative performance of genotypic values.
