# Supplementary material for: Importance of mega-environments in evaluation and identification of climate resilient maize hybrids (Zea mays L.)
Source: PLoS One. 2023 Dec 14;18(12):e0295518. doi: 10.1371/journal.pone.0295518 (PMC10721017; doi:10.1371/journal.pone.0295518)
Supplement: S4 Table — (PDF) [file pone.0295518.s004.pdf]

S4 Table. Ranking of hybrids based on AMMI and WAASB stability indices

2016																			
Hybrid	GY	Y_R	ASV	ASV_R	ASV_SSI	SIPC	SIPC_R	SIPC_SSI	EV	EV_R	EV_SSI	ZA	ZA_R	ZA_SSI	WAAS	WAAS_R	WAAS_SSI	WAASY	OrWAASY
G1	6450	5	2.25	7	12	6.98	10	15	0.07	10	15	0.19	8	13	1.31	8	13	62.15	5
G10	6392	7	2.93	8	15	8.27	12	19	0.09	11	18	0.22	11	18	1.61	11	18	52.47	9
G11	5627	11	1.16	2	13	3.58	2	13	0.02	1	12	0.09	2	13	0.67	2	13	60.88	7
G12	5029	15	5.37	13	28	5.92	9	24	0.05	8	23	0.24	12	27	1.95	13	28	11.65	15
G13	6310	9	3.98	12	21	9.66	14	23	0.14	14	23	0.26	13	22	1.91	12	21	42.21	11
G14	7204	1	3.09	9	10	4.28	4	5	0.03	4	5	0.14	5	6	1.12	5	6	84.58	1
G15	6393	6	2.21	5	11	5.69	7	13	0.06	9	15	0.17	6	12	1.28	7	13	61.72	6
G2	6713	4	2.23	6	10	3.97	3	7	0.03	3	7	0.12	4	8	0.89	4	8	79.76	2
G3	5452	12	3.5	10	22	5.71	8	20	0.04	6	18	0.2	10	22	1.51	10	22	33.48	13
G4	6384	8	5.61	14	22	10.19	15	23	0.16	15	23	0.32	15	23	2.37	15	23	31.14	14
G5	5406	13	3.67	11	24	5.58	6	19	0.04	5	18	0.19	9	22	1.47	9	22	33.67	12
G6	5348	14	1.09	1	15	4.74	5	19	0.04	7	21	0.11	3	17	0.74	3	17	52.52	8
G7	6842	3	2.01	4	7	7.13	11	14	0.1	12	15	0.18	7	10	1.22	6	9	73.63	3
G8	5919	10	1.18	3	13	2.81	1	11	0.02	2	12	0.08	1	11	0.57	1	11	70.45	4
G9	6862	2	6.28	15	17	8.49	13	15	0.11	13	15	0.29	14	16	2.26	14	16	45.27	10
2017																			
G1	6352	11	2.4	4	15	3.46	5	16	0.03	5	16	0.14	3	14	1.19	3	14	46.84	7
G10	7596	3	4.39	11	14	7.61	15	18	0.15	15	18	0.28	13	16	2.3	12	15	54.17	5
G11	7529	5	0.46	1	6	0.86	1	6	0	1	6	0.03	1	6	0.26	1	6	92.83	2
G12	5705	15	2.16	3	18	5.13	8	23	0.09	11	26	0.16	6	21	1.3	6	21	29.52	12
G13	6135	13	3.95	9	22	5.34	9	22	0.06	8	21	0.22	9	22	1.88	10	23	28.05	15
G14	6577	9	4.7	12	21	7.04	13	22	0.11	13	22	0.28	14	23	2.38	13	22	28.61	14
G15	6020	14	3.68	8	22	4.65	6	20	0.06	7	21	0.2	7	21	1.69	7	21	29.09	13
G2	6519	10	3.63	7	17	2.77	3	13	0.02	4	14	0.14	4	14	1.28	4	14	48.93	6
G3	7834	1	3.43	6	7	6	11	12	0.1	12	13	0.22	10	11	1.87	9	10	68.17	3
G4	7297	6	4.36	10	16	4.66	7	13	0.05	6	12	0.2	8	14	1.79	8	14	57.25	4
G5	6735	8	5.64	14	22	5.4	10	18	0.07	9	17	0.25	11	19	2.27	11	19	34.54	11
G6	7592	4	6.43	15	19	7.58	14	18	0.13	14	18	0.32	15	19	2.79	15	19	44.32	8
G7	7739	2	0.53	2	4	1.06	2	4	0	2	4	0.04	2	4	0.31	2	4	96.85	1
G8	6867	7	5.31	13	20	6.36	12	19	0.09	10	17	0.27	12	19	2.42	14	21	34.71	10
G9	6297	12	3.23	5	17	3.04	4	16	0.02	3	15	0.14	5	17	1.29	5	17	43.62	9

**2018**																			
G1	8566	7	2.27	3	10	5.33	5	12	0.05	4	11	0.15	3	10	1.15	3	10	67.04	4
G10	8316	9	0.86	2	11	1.75	1	10	0.01	1	10	0.05	1	10	0.38	1	10	77.53	2
G11	9024	3	8.56	10	13	9.49	10	13	0.13	10	13	0.34	10	13	2.83	10	13	42.89	10
G12	9565	2	7.1	9	11	9.37	9	11	0.12	8	10	0.33	9	11	2.65	9	11	54.93	7
G2	7487	10	3.57	6	16	4.61	3	13	0.04	3	13	0.16	4	14	1.26	4	14	47.83	8
G3	6590	12	8.89	11	23	9.95	12	24	0.14	12	24	0.37	12	24	3.05	12	24	0	12
G4	8602	6	5.28	8	14	9.69	11	17	0.14	11	17	0.3	8	14	2.34	8	14	45.4	9
G5	9725	1	5.2	7	8	3.82	2	3	0.02	2	3	0.17	5	6	1.45	7	8	80.02	1
G6	8652	5	0.56	1	6	5.04	4	9	0.07	5	10	0.11	2	7	0.75	2	7	76.1	3
G7	8485	8	2.82	5	13	6.34	6	14	0.08	7	15	0.18	7	15	1.36	6	14	61.85	6
G8	8719	4	2.46	4	8	6.53	7	11	0.08	6	10	0.18	6	10	1.29	5	9	67.02	5
G9	7272	11	9.16	12	23	9.02	8	19	0.13	9	20	0.36	11	22	3.03	11	22	11.32	11
**2019**																			
G1	6720	9	1.67	3	12	2.76	2	11	0.02	1	10	0.12	2	11	0.97	2	11	59.3	3
G10	7990	2	3.68	11	13	6.19	10	12	0.15	12	14	0.25	9	11	1.99	9	11	59.56	2
G11	7420	6	3.57	9	15	6.12	9	15	0.13	9	15	0.25	10	16	2.02	10	16	45.84	9
G12	7430	5	2.58	4	9	5.65	8	13	0.1	8	13	0.22	8	13	1.76	8	13	53.2	5
G2	7470	4	0.692	1	5	2.42	1	5	0.03	2	6	0.08	1	5	0.66	1	5	85.21	1
G3	8120	1	4.28	12	13	6.63	12	13	0.13	10	11	0.29	12	13	2.44	12	13	50	6
G4	5910	12	3.6	10	22	4.67	5	17	0.1	7	19	0.2	6	18	1.67	7	19	21.44	12
G5	6230	11	1.65	2	13	3.12	3	14	0.03	3	14	0.12	3	14	0.99	3	14	47.81	8
G6	7130	7	2.66	5	12	4.69	6	13	0.06	5	12	0.2	5	12	1.66	5	12	49.28	7
G7	7560	3	3.02	8	11	4.87	7	10	0.08	6	9	0.2	7	10	1.67	6	9	58.83	4
G8	6450	10	2.76	6	16	4.31	4	14	0.05	4	14	0.2	4	14	1.64	4	14	34.65	10
G9	6850	8	3	7	15	6.6	11	19	0.13	11	19	0.26	11	19	2.08	11	19	31.19	11

Note: GY – Grain yield (kg/ha), R – Rank of the given parameter, SSI - simultaneous selection indexes, ASV - AMMI-stability value, SIPC - Sums of the Absolute Value of the IPC Scores,EV - Eigenvector Values, ZA - Absolute Value of the Relative Contribution of IPCs to the Interaction, WAAS - Weighted average of absolute scores, WAASY - Weighting between stability and mean performance, OrWAASY - Rank for the WAASY value
