# Supplementary material for: Importance of mega-environments in evaluation and identification of climate resilient maize hybrids (Zea mays L.)
Source: PLoS One. 2023 Dec 14;18(12):e0295518. doi: 10.1371/journal.pone.0295518 (PMC10721017; doi:10.1371/journal.pone.0295518)
Supplement: S1 Appendix — (PDF) [file pone.0295518.s005.pdf]

S1 Appendix. R code for analysis of individual ANOVA and combined ANOVA

```
### Import Dataset
library(readxl) # load libraries
file<- read_excel("~/datafile.xlsx")
View(file)
### The data has four Columns ENV, GEN, REP and GY.
#################### ANOVA Analysis ##########################
library(metan) # load libraries
### Individual ANOVA
indiv<- anova_ind(file, ENV, GEN, REP, GY)
capture.output(indiv, file = "Individual ANOVA.txt")
### Combined ANOVA
joint <- anova_joint(file, ENV, GEN, REP, GY)
capture.output(joint, file = "Joint ANOVA.txt")

### Calculation of Correlation between stability indexes
library(metan) # load libraries
library(ggplot2)# load libraries
model <- ge_stats(file, ENV, GEN, REP, GY)
c <- corr_stab_ind(model, stats = c("Y, ASV, ZA, SIPC, EV, HMRPGV, HMGV, RPGV, WAAS"))
### Save the plot
ggsave("Correlation between stability indexes.jpeg",
    plot = c,
    width = 5, height = 5,
    units = "in",
    dpi = 300)
```
