# Supplementary material for: Importance of mega-environments in evaluation and identification of climate resilient maize hybrids (Zea mays L.)
Source: PLoS One. 2023 Dec 14;18(12):e0295518. doi: 10.1371/journal.pone.0295518 (PMC10721017; doi:10.1371/journal.pone.0295518)
Supplement: S2 Appendix — (PDF) [file pone.0295518.s006.pdf]

S2 Appendix. R code for estimation of stability indices

```
### AMMI indices

# First generate AMMI model

library(metan)

AMMI_model<- performs_ammi(file, ENV, GEN, REP, GY, verbose = FALSE)

# Significant axis used for prediction

predicted <- predict(AMMI_model)

# Estimate AMMI stability indices

stab_indexes<- AMMI_indexes(AMMI_model)

# Plot biplot

plot_scores(AMMI_model) # GY x PC1 biplot

plot_scores(AMMI_model, type = 2) # PC1 x PC2 biplot

plot_scores(AMMI_model, type = 4) # Nominal yield x Environment PC1


### WAAS biplot

waas_index<- waas(file,  ENV, GEN, REP, GY, verbose = FALSE)


### Fitting mixed model to estimate BLUP indices

mixed_mod<-   gamem_met(file,
          env = ENV,
          gen = GEN,
          rep = REP,
resp = everything(),
          random = "all", #Other options gen, env and all.
          verbose = TRUE)
### BLUP based stability index
# Fit WAASB model
plot_scores(waasb_model, type = 1) # GY x PC1 biplot
plot_scores(waasb_model, type = 4) # Nominal yield x Environment PC1 plot
waasb_model<- waasb(file, ENV, GEN, REP, GY, verbose = FALSE)
# BLUP stability indices
blup_indexes<- blup_indexes(waasb_model)


### GGE biplot analysis

gge_model<- gge(file, ENV, GEN, GY, svp = "environment") # GGE model

plot(gge_model, type = 3) # Which won where biplot
```
